# Changes in Bone Microarchitecture and Inflammatory Cytokines After Cure of Chronic Hepatitis C Infection With Direct-Acting Antiviral Therapy

**DOI:** 10.1093/ofid/ofaf571

**Published:** 2025-09-15

**Authors:** Vincent Lo Re, Dean M Carbonari, Craig W Newcomb, Jessie Torgersen, Erica J Weinstein, Shanae M Smith, Katherine L Brecker, X Sherry Liu, Jay R Kostman, Stacey Trooskin, Rebecca A Hubbard, Joshua F Baker, Babette S Zemel, Mary B Leonard

**Affiliations:** Division of Infectious Diseases, Department of Medicine, Perelman School of Medicine, University of Pennsylvania, Philadelphia, Pennsylvania, USA; Center for Clinical Epidemiology and Biostatistics, Center for Real-world Effectiveness and Safety of Therapeutics, Department of Biostatistics, Epidemiology, and Informatics, Perelman School of Medicine, University of Pennsylvania, Philadelphia, Pennsylvania, USA; Center for Clinical Epidemiology and Biostatistics, Center for Real-world Effectiveness and Safety of Therapeutics, Department of Biostatistics, Epidemiology, and Informatics, Perelman School of Medicine, University of Pennsylvania, Philadelphia, Pennsylvania, USA; Center for Clinical Epidemiology and Biostatistics, Center for Real-world Effectiveness and Safety of Therapeutics, Department of Biostatistics, Epidemiology, and Informatics, Perelman School of Medicine, University of Pennsylvania, Philadelphia, Pennsylvania, USA; Division of Infectious Diseases, Department of Medicine, Perelman School of Medicine, University of Pennsylvania, Philadelphia, Pennsylvania, USA; Center for Clinical Epidemiology and Biostatistics, Center for Real-world Effectiveness and Safety of Therapeutics, Department of Biostatistics, Epidemiology, and Informatics, Perelman School of Medicine, University of Pennsylvania, Philadelphia, Pennsylvania, USA; Division of Infectious Diseases, Department of Medicine, Perelman School of Medicine, University of Pennsylvania, Philadelphia, Pennsylvania, USA; Center for Clinical Epidemiology and Biostatistics, Center for Real-world Effectiveness and Safety of Therapeutics, Department of Biostatistics, Epidemiology, and Informatics, Perelman School of Medicine, University of Pennsylvania, Philadelphia, Pennsylvania, USA; Center for Clinical Epidemiology and Biostatistics, Center for Real-world Effectiveness and Safety of Therapeutics, Department of Biostatistics, Epidemiology, and Informatics, Perelman School of Medicine, University of Pennsylvania, Philadelphia, Pennsylvania, USA; Department of Orthopedic Surgery, Perelman School of Medicine, University of Pennsylvania, Philadelphia, Pennsylvania, USA; Department of Bioengineering, University of Pennsylvania, Philadelphia, Pennsylvania, USA; Philadelphia FIGHT, Philadelphia, Pennsylvania, USA; Mazzoni Center, Philadelphia, Pennsylvania USA; Department of Biostatistics, School of Public Health, Brown University, Providence, Rhode Island, USA; Division of Rheumatology, Department of Medicine, Perelman School of Medicine, University of Pennsylvania, Philadelphia, Pennsylvania, USA; Division of Gastroenterology, Hepatology, and Nutrition, Department of Pediatrics, Children's Hospital of Philadelphia, University of Pennsylvania Perelman School of Medicine, Philadelphia, Pennsylvania, USA; Department of Pediatrics and Medicine, Stanford University School of Medicine, Palo Alto, California, USA

**Keywords:** bone, cytokines, dual-energy X-ray absorptiometry, hepatitis C virus, peripheral quantitative computed tomography

## Abstract

**Background:**

It remains unclear if cure of hepatitis C virus (HCV) infection with direct-acting antivirals (DAAs) ameliorates HCV-related inflammation and bone deficits. We evaluated changes in cytokines and bone measurements by high-resolution peripheral quantitative computed tomography (HR-pQCT) prior to DAA treatment and 18 months following initiation and compared changes in uninfected controls over 18 months.

**Methods:**

We conducted a cohort study of 40 participants who initiated DAAs and achieved cure and 48 without HCV as controls. At enrollment and 18 months later, participants had measurements of volumetric bone mineral density, cortical dimensions, and mechanical properties of the radius and tibia by HR-pQCT; visceral fat area and appendicular lean mass by whole-body dual-energy X-ray absorptiometry; and serum tumor necrosis factor alpha (TNF-α), interleukin 6 (IL-6), and interleukin 18 (IL-18). Multivariable linear regression was used to estimate group differences in mean changes in bone measurements and cytokines.

**Results:**

We observed no significant differences in month 0–18 changes in HR-pQCT measurements between participants with cured HCV and controls in unadjusted models or after adjustment for age, sex, appendicular lean mass index, visceral fat area, and smoking. Participants with cured HCV had decreases in IL-18 (mean change, −0.085 vs +0.086 log pg/mL; *P* < .001) and TNF-α (mean change, −0.050 vs +0.084 log pg/mL; *P* < .001), but not IL-6 (mean change, +0.108 vs +0.009 log pg/mL; *P* = .214) versus controls.

**Conclusions:**

Participants with cured HCV had no significant changes in bone microarchitecture by HR-pQCT 18 months after DAA initiation compared with controls, but did have decreases in IL-18 and TNF-α versus controls.

## Graphical Abstract

**Graphical Abstract:**
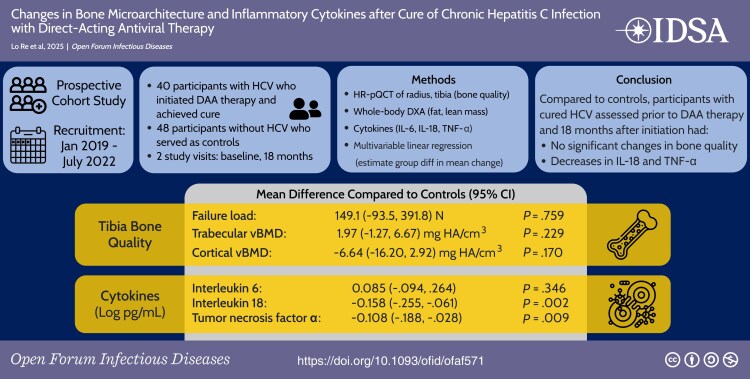
The graphical abstract is also available at Tidbit: https://tidbitapp.io/tidbits/changes-in-bone-microarchitecture-and-inflammatory-cytokines-after-cure-of-chronic-hepatitis-c-infection-with-direct-acting-antiviral-therapy?utm_campaign=tidbitlinkshare&amp;utm_source=IO

In addition to its effects on the liver, chronic hepatitis C virus (HCV) infection can have extrahepatic effects [1], including on bone, which has been referred to as “hepatic osteodystrophy” [2, 3]. Cross-sectional studies have shown that bone mineral density (BMD) as determined by dual-energy X-ray absorptiometry (DXA) is lower in people with chronic HCV than those without HCV infection [4–7]. Cohort studies have demonstrated that chronic HCV infection is also associated with increased rates of bone fractures [7–9], particularly at the hip, which can adversely affect survival, with an effect on mortality similar to that of cardiovascular disease [10]. Consequently, people with chronic HCV infection, especially those with other risk factors for osteoporosis, may possibly benefit from BMD screening by DXA to identify bone loss.

We recently used high-resolution peripheral quantitative computed tomography (HR-pQCT), a 3-dimensional X-ray–based imaging technique that can discriminate between trabecular and cortical bone when estimating volumetric BMD [11, 12], to show that patients with chronic HCV infection had lower radius and tibia trabecular volumetric BMD and tibia cortical dimensions compared to people without HCV, independent of age, sex, visceral fat area, appendicular lean mass, and smoking [13]. Chronic HCV stimulates production of inflammatory cytokines, such as tumor necrosis factor alpha (TNF-α), interleukin 6 (IL-6), and interleukin 18 (IL-18) [14–18], which can promote low BMD by inhibiting osteoblasts (decreasing bone formation) [19–22] and stimulating osteoclasts (increasing bone resorption) [23, 24]. We found that higher log TNF-α levels were associated with lower radius trabecular volumetric BMD, lower tibia cortical volumetric BMD, and higher tibia cortical porosity by HR-pQCT [13], suggesting that HCV-associated inflammation might be an important contributor to bone deficits.

Given the adverse impact that chronic HCV-related inflammation might have on bone microarchitecture, cure of chronic HCV may halt or reverse these detrimental effects. Direct-acting antiviral (DAA) therapy achieves HCV cure in ≥94% of patients with ≤12 weeks of treatment [25–33]. However, the extent to which HCV-related cytokines are reduced, and bone deficits are improved, following cure remains unclear. Evaluating changes in bone microarchitecture and cytokine levels following HCV cure might yield important information on the reversibility of HCV-related inflammation and bone abnormalities and could identify important determinants of improvements in bone health in people with chronic HCV infection.

To address this knowledge gap, we evaluated changes in inflammatory cytokines and bone measurements by HR-pQCT and DXA following cure of chronic HCV with DAA therapy. We examined participants who initiated DAA therapy and achieved cure. We measured cytokines and bone outcomes prior to treatment and 18 months later (approximately 1 year following HCV cure). We compared results to participants without HCV with the same measurements assessed at enrollment and 18 months later. We hypothesized that HCV cure would result in decreases in cytokine levels and increases in bone quality and mechanical properties 18 months after DAA treatment initiation and that these changes would be greater than those without HCV over a similar 18-month period.

## METHODS

### Study Design and Setting

We performed a prospective cohort study of DAA-naive individuals with chronic HCV and those without HCV. Patients with chronic HCV were recruited from viral hepatitis and primary care practices within the University of Pennsylvania Health System and from Philadelphia FIGHT. Primary care practices were the recruitment sites for participants without HCV. Study personnel recruited individuals who presented to their primary care provider for routine care visits to serve as controls. All clinics were located in Philadelphia, Pennsylvania.

Patients scheduled for clinical visits were prescreened for HCV infection and, after approval from their practitioner, approached by study personnel for interest in participation. Self-reported responses were used to assess the potential eligibility of interested patients. Eligibility was confirmed by medical record review after obtaining informed consent. We used stratified sampling to recruit patients without HCV according to sex assigned at birth, race (Black vs not Black), and age category (18–39, 40–59, ≥60 years) to promote balance in demographic characteristics between cohorts. Participants were recruited from 1 January 2019 through 31 July 2022.

After enrollment, all participants underwent a baseline (month 0) assessment of demographic and clinical characteristics, height and body weight, bone measurements via HR-pQCT and DXA, and cytokines. At follow-up (month 18), HR-pQCT, DXA, and cytokine assessments were repeated. Cure of HCV was defined as most recent HCV RNA undetectable.

### Patient Consent Statement

The study protocol was approved by the University of Pennsylvania Institutional Review Board (IRB). The Philadelphia FIGHT IRB relied on the University of Pennsylvania IRB through a reliance agreement. Written informed consent was provided by all participants.

### Study Participants

Patients with chronic HCV were eligible if they were ≥18 years of age, had detectable HCV RNA, and underwent liver fibrosis staging within the past 6 months. Human immunodeficiency virus (HIV) coinfection was allowed if the participant was on a stable antiretroviral therapy regimen for ≥4 weeks before enrollment with documented HIV RNA <200 copies/mL. Patients without HCV (controls) were eligible if they were ≥18 years of age and HCV and HIV uninfected. At both study visits, controls were confirmed to be HCV and HIV antibody-negative by OraSure's OraQuick assay.

Patients were excluded if conditions that might affect bone were present, including chronic kidney disease (estimated glomerular filtration rate <60 mL/minute/1.73 m^2^) [34]; cancer (excluding non-melanomatous skin malignancy); malabsorption (due to celiac disease, small bowel resection surgery, chronic diarrhea); weight loss >5% of body weight over the previous 3 months; or another chronic liver disease (ie, hepatitis B, hemochromatosis, alpha-1-antitrypsin, autoimmune hepatitis, sclerosing cholangitis, biliary cirrhosis, Wilson disease, metabolic dysfunction–associated steatotic liver disease). We also excluded patients who were pregnant (to avoid DXA radiation) or had prior bilateral lower leg fractures. Patients with chronic HCV were excluded if they had HCV genotype 3 infection (which promotes hepatic steatosis [35] and low BMD [36]) or prior HCV therapy (which might affect BMD) [37].

### Assessment of Demographic, Clinical, and Anthropometric Data

Data collected at baseline included age; sex assigned at birth; race and ethnicity; current smoking; alcohol consumption in the past year determined by the 10-item Alcohol Use Disorders Identification Test [38]; drug use in the past year determined by the 10-item Drug Abuse Screening Test [39]; history of injection drug use; fracture history; calcium and vitamin D use; and, if applicable, postmenopausal status, defined by either: (1) absence of menstrual periods for >12 months in a previously menstruating individual >45 years of age while not on hormonal birth control, or (2) prior hysterectomy or bilateral oophorectomy.

Data collected from all participants’ medical records within 12 months prior to enrollment included diabetes mellitus (defined by diagnosis, hemoglobin A1c ≥6.5%, or random glucose ≥200 mg/dL) and serum creatinine. Among participants with chronic HCV, we reviewed records to obtain HCV diagnosis date, most recent pretreatment HCV RNA/genotype, and pretreatment liver fibrosis stage by vibration-controlled transient elastography or HCV FibroSure test [40]. Advanced hepatic fibrosis/cirrhosis was defined as METAVIR stage F3 or F4 [40].

Body weight and height were measured at months 0 and 18 in triplicate without shoes using a digital scale (Scaltronix) and stadiometer (Holtain), respectively, and the mean of each was used to calculate body mass index.

### HR-pQCT Measurements

Radius and tibia trabecular and cortical bone measurements were obtained by HR-pQCT (Xtreme CT II; SCANCO Medical AG) at months 0 and 18. The nondominant side was used unless there was a previous fracture or interfering artifact. A scout view was used to place the reference line at the distal epiphysis/endplate. Bone measurements were obtained at 4% of the radius and 7.3% of the tibia lengths (ultradistal) and 30% (midshaft) of the radius and tibia lengths proximal to the reference line. Scans were analyzed for trabecular volumetric BMD (measured in mg hydroxyapatite [HA]/cm^3^) at the ultradistal site and cortical measures at the midshaft site. Cortical volumetric BMD (mg HA/cm^3^) was assessed after exclusion of pore space and surface voxels to minimize partial volume voxels. Cortical porosity (%) was calculated as the ratio of intracortical pore volume to total volume of the cortical compartment [41]. Cortical area (mm^2^), thickness (mm), and perimeter (mm) were calculated using the direct 3D distance transformation method [42]. Cortical pore diameter (mm) was measured by direct 3D method [43]. Tibia/radius bone mechanical properties, including axial stiffness (Newtons [N]/mm) and failure load (N), were determined by micro-finite element analysis (SCANCO Medical AG) [44–48].

### DXA Measurements

Areal BMD (g/cm^2^) at the total hip, femoral neck, and lumbar (L1–L4) spine; L1–L4 trabecular bone score; visceral fat area (cm^2^); whole-body fat (kg); and appendicular lean mass (kg) were assessed using a Delphi (Hologic) and/or Horizon (Hologic) bone densitometer at months 0 and 18. Two densitometers were used because the Delphi was replaced by the Horizon during the study. Quality control was monitored weekly using a phantom. Appendicular lean mass, a measure of skeletal muscle [49], and whole-body fat mass were converted to appendicular lean mass index (ALMI; kg/m^2^) and fat mass index (FMI; kg/m^2^) using height.

### Laboratory Data

Serum levels of IL-6, IL-18, and TNF-α (all in pg/mL) were measured using an enzyme-linked immunosorbent assay (Ella; ProteinSimple, Bio-Techne). The testing of the inflammatory cytokines was conducted in the same laboratory and run in 3 batches over the course of the study. The laboratory ran controls for each inflammatory cytokine with each batch of samples.

### Statistical Analysis

To assess whether cure of HCV with DAA therapy affected bone outcomes, we assessed the mean changes in HR-pQCT and DXA measurements from month 0 to month 18 in the 2 cohorts. We used multivariable linear regression to estimate mean differences (95% confidence intervals [CIs]) between the groups in month 0–18 changes in bone measurements, after adjustment for age (continuous), sex, change in ALMI by DXA (continuous), change in visceral fat area by DXA (continuous), and smoking at month 0. We adjusted for ALMI and visceral fat area because bone quality is associated with muscle mass [50, 51] and visceral fat mass [52–61]. Since the DXA scanner was replaced during the study, we additionally adjusted analyses of DXA outcomes for a variable indicating that different machines were used by the participant. To assess the robustness of the results, we repeated analyses replacing change in visceral fat area with change in FMI (continuous) by DXA.

To explore the impact of HCV cure on bone outcomes among participants with HIV/HCV coinfection, we repeated the analysis, stratifying the HCV cohort by HIV coinfection status. We used multivariable linear regression to estimate mean differences (95% CI) in month 0–18 changes in bone outcomes between the control group and participants with: (1) HIV/HCV coinfection, and (2) HCV alone, after adjustment for age (continuous), sex, change in ALMI (continuous), change in visceral fat area (continuous), and smoking.

Next, to evaluate the effect of cure on cytokine levels, we estimated month 0–18 changes in mean log IL-6, IL-18, and TNF-α between the cohorts. We used multivariable linear regression to estimate mean differences (95% CIs) between the groups in changes in log cytokine levels, after adjustment for age (continuous), sex, change in visceral fat area (continuous), and smoking. We adjusted for visceral fat because this contributes to secretion of cytokines [62–66]. Cytokine levels were log transformed because of a more linear relationship with continuous covariates after transformation. We repeated analyses replacing change in visceral fat area with change in FMI (continuous) to assess the robustness of the results.

We examined scatterplots to confirm the linearity of relationships between outcomes and continuous predictors. Analyses were conducted using SAS version 9.4 software (SAS Institute).

## RESULTS

### Participant Characteristics

**Figure 1.**
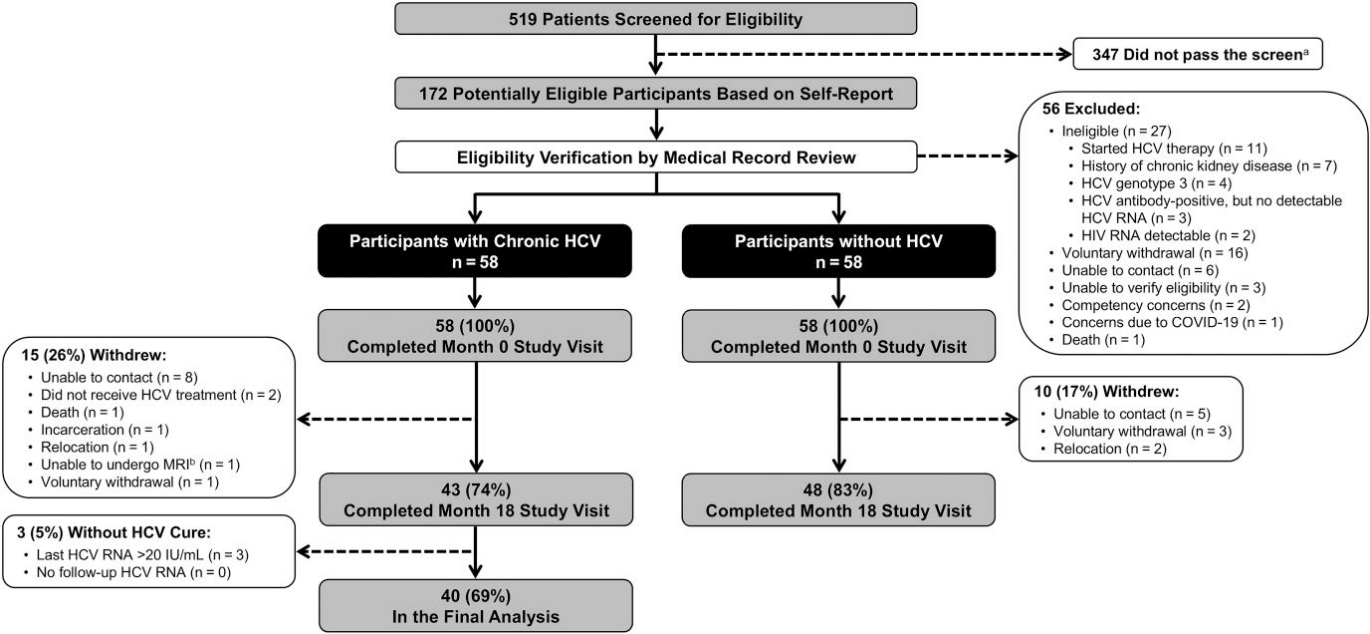
Cohort selection flowchart. ^a^Screening questions have been previously published [13]. ^b^This participant was withdrawn before the MRI component was made optional. Abbreviations: COVID-19, coronavirus disease 2019; HCV, hepatitis C virus; HIV, human immunodeficiency virus; MRI, magnetic resonance imaging.

Among 519 patients screened for eligibility, 172 were potentially eligible based on self-report; after medical record review, 56 were excluded (reasons reported in Figure 1). The month 0 visit was completed by 116 participants (58 with chronic HCV; 58 without HCV). After month 0, 15 participants with chronic HCV and 10 participants without HCV withdrew from the study (reasons reported in Figure 1). All remaining participants completed the month 18 study visit. Among the remaining 43 participants with chronic HCV, 3 (7%) did not achieve cure and were excluded. A total of 98 participants (40 with chronic HCV; 48 without HCV) were included in the final analysis.

Participants with chronic HCV were older, more likely to smoke, and more frequently reported injection drug use within the past year (Table 1). No other significant differences in demographic or clinical characteristics between the groups were observed. Ten participants with chronic HCV had advanced hepatic fibrosis/cirrhosis. Twelve (30%) of the participants with chronic HCV also had HIV coinfection with undetectable HIV RNA on stable antiretroviral therapy (11 on a regimen containing tenofovir alafenamide; 1 on dolutegravir/abacavir/lamivudine).

**Table 1.** Baseline Characteristics of Participants in the Final Analysis, by Hepatitis C Status

Characteristic	Participants With HCV (n = 40)	Participants Without HCV (n = 48)	*P* Value
Recruitment site			
University of Pennsylvania Health System	33 (82.5)	37 (77.1)	
Philadelphia FIGHT	7 (17.5)	0 (0.0)	
Referral	0 (0.0)	11 (22.9)	
Age, y, median (IQR)	60 (45–64)	54 (37–63)	.**011**
Missing	0 (0.0)	0 (0.0)	
Male sex	31 (77.5)	31 (64.6)	.186
Missing	0 (0.0)	0 (0.0)	
Race			.174
Asian	0 (0)	2 (4.2)	
Black or African American	30 (75.0)	28 (58.3)	
White	9 (22.5)	16 (33.3)	
Other	1 (2.5)	2 (4.2)	
Missing	0 (0.0)	0 (0.0)	
Hispanic ethnicity	2 (5.0)	3 (6.3)	.801
Missing	0 (0.0)	0 (0.0)	
Body mass index, kg/m^2^			
Median (IQR)	28 (25–30)	28 (24–33)	.202
Underweight (<18.5)	0 (0)	1 (2.1)	.419
Normal (18.5–24.9)	13 (32.5)	12 (25.0)	
Overweight (25.0–29.9)	15 (37.5)	14 (29.2)	
Obesity (≥30.0)	12 (30.0)	21 (43.8)	
Missing	0 (0.0)	0 (0.0)	
Diabetes mellitus^a^	12 (30.0)	10 (20.8)	.323
Missing	0 (0.0)	0 (0.0)	
HIV coinfection	12 (30)	0 (0)	**<**.**001**
Serum creatinine, mg/dL, median (IQR)^b^	0.95 (0.83–1.07)	0.90 (0.78–1.06)	.488
Missing	1 (2.5)	13 (27.1)	.**002**
Total cholesterol, mg/dL, median (IQR)	154 (135–182)	169 (147–193)	.269
Missing	3 (7.5)	1 (2.1)	.225
HDL cholesterol, mg/dL, median (IQR)	49 (41–60)	51 (45–65)	.275
Missing	3 (7.5)	1 (2.1)	.225
LDL cholesterol, mg/dL, median (IQR)	91 (76–111)	93 (72–120)	.827
Missing	3 (7.5)	1 (2.1)	.225
Triglycerides, mg/dL, median (IQR)	105 (79–129)	90 (64–135)	.658
Missing	3 (7.5)	1 (2.1)	.225
Dyslipidemia^c^	15 (37.5)	18 (37.5)	>.999
Missing	0 (0.0)	0 (0.0)	
History of bone fracture	23 (57.5)	21 (43.8)	.199
Missing	0 (0.0)	0 (0.0)	
Calcium supplement use	5 (12.5)	5 (10.4)	.759
Missing	0 (0.0)	0 (0.0)	
Vitamin D supplement use	8 (20.0)	13 (27.1)	.438
Missing	0 (0.0)	0 (0.0)	
Postmenopausal (females only)^d^	4/9 (44.4)	7/17 (41.2)	.873
Missing	0 (0.0)	0 (0.0)	
Smoking status			
Never	4 (10.0)	26 (54.2)	**<**.**001**
Former	9 (22.5)	9 (18.8)	
Current	27 (67.5)	13 (27.1)	
Missing	0 (0.0)	0 (0.0)	
Smoking pack-years, median (IQR)^e^	8.75 (4.80–12.00)	5.00 (2.15–13.50)	.739
Missing	0 (0.0)	0 (0.0)	
Self-reported history of injection drug use	18 (45.0)	0 (0)	**<**.**001**
Time since initial HCV diagnosis^f^			
<5 y	22 (55.0)	…	
5–10 y	8 (20.0)	…	
11–20 y	6 (15.0)	…	
21–40 y	3 (7.5)	…	
>40 y	1 (2.5)	…	
Alcohol use in past year, AUDIT score			
Median (IQR)	2 (0–5)	2 (1–5)	.805
<8 (low risk)	34 (85.0)	43 (89.6)	.803
8–15 (risky/hazardous)	5 (12.5)	4 (8.3)	
16–19 (high-risk)	0 (0.0)	0 (0.0)	
≥20 (highest risk)	1 (2.5)	1 (2.1)	
Missing	0 (0.0)	0 (0.0)	
Drug use in past year, DAST-10 score			
Median (IQR)	1 (0–5)	0 (0–1)	.**028**
0 (none)	20 (50.0)	35 (72.9)	.**004**
1–2 (low)	7 (17.5)	12 (25.0)	
3–5 (moderate)	4 (10.0)	1 (2.1)	
6–8 (substantial)	6 (15.0)	0 (0)	
9–10 (severe)	3 (7.5)	0 (0)	
Missing	0 (0.0)	0 (0.0)	

Data are reported as No. (%) unless otherwise noted. Bold indicates *P* < .05.

Abbreviations: AUDIT, Alcohol Use Disorders Identification Test; DAST-10, 10-item Drug Abuse Screening Test; HCV, hepatitis C virus; HDL, high-density lipoprotein; HIV, human immunodeficiency virus; IQR, interquartile range; LDL, low-density lipoprotein.

^a^Diabetes mellitus was defined as the presence of any of the following: diagnosis of diabetes in the medical record, hemoglobin A1c ≥6.5%, or random glucose ≥200 mg/dL.

^b^Serum creatinine levels from the prior 12 months, if available, were collected from the medical record.

^c^Dyslipidemia was defined as triglycerides ≥150 mg/dL or HDL cholesterol <40 mg/dL (if male) or <50 mg/dL (if female).

^d^Postmenopausal status (if applicable) defined as either the absence of menstrual periods for >12 months in a previously menstruating individual >45 years of age or status post hysterectomy or bilateral oophorectomy.

^e^For current smokers, smoking pack-years was calculated as the average number of cigarettes smoked per day divided by 20, then multiplied by the number of years the person has smoked.

^f^Time since initial HCV diagnosis based on the self-reported year of initial HCV diagnosis.

### Month 0–18 Changes in HR-pQCT and DXA Measurements

We observed no statistically significant differences in mean change in any HR-pQCT measures of the radius or tibia from month 0 to month 18 between participants with cured HCV and those without HCV prior to adjustment (Table 2, Supplementary Table 1) or after adjustment for age, sex, change in ALMI, change in visceral fat area, and smoking. Results were similar when change in visceral fat area was replaced with change in FMI (Supplementary Table 2).

**Table 2.** Unadjusted Mean Change in High-Resolution Peripheral Quantitative Computed Tomography, Dual-Energy X-Ray Absorptiometry, and Laboratory Measurements From Month 0 to Month 18 in Participants With and Without Hepatitis C Virus

Measurement	Participants With HCV (n = 40)			Participants Without HCV (n = 48)			Between-Group Difference (95% CI)	*P* Value for Diff in Change
	Mean (SD) at Month 0	Mean (SD) at Month 18	Mean (SD) Change	Mean (SD) at Month 0	Mean (SD) at Month 18	Mean (SD) Change		
HR-pQCT of radius^a^								
Total vBMD (mg HA/cm^3^)	306.727 (76.49)	309.632 (76.23)	2.905 (16.68)	353.222 (65.13)	356.372 (64.57)	3.150 (8.79)	−0.245 (−5.914, 5.425)	.932
Trabecular vBMD (mg HA/cm^3^)	155.492 (45.43)	159.124 (48.01)	3.632 (16.44)	179.483 (38.37)	180.133 (38.44)	0.650 (4.60)	2.982 (−2.065, 8.029)	.243
Cortical vBMD (mg HA/cm^3^)	1073.024 (36.38)	1071.658 (37.02)	−1.366 (13.63)	1088.689 (28.50)	1087.691 (28.71)	−0.998 (9.13)	−0.368 (−5.370, 4.634)	.884
Cortical area (mm^2^)	99.721 (18.55)	99.711 (19.23)	−0.011 (3.16)	96.920 (15.35)	96.796 (16.01)	−0.124 (2.53)	0.114 (−1.128, 1.356)	.856
Cortical porosity (%)	0.511 (0.47)	0.511 (0.43)	0.000 (0.24)	0.316 (0.26)	0.347 (0.32)	0.031 (0.18)	−0.031 (−.122, .060)	.499
Cortical perimeter (mm)	45.218 (4.35)	45.126 (4.40)	−0.092 (0.75)	42.991 (3.88)	43.062 (3.94)	0.071 (0.45)	−0.163 (−.429, .103)	.226
Cortical thickness (mm)	3.347 (0.53)	3.340 (0.57)	−0.007 (0.16)	3.512 (0.43)	3.496 (0.45)	−0.017 (0.13)	0.010 (−.053, .073)	.758
Cortical pore diameter (mm)	0.164 (0.04)	0.170 (0.06)	0.006 (0.06)	0.159 (0.04)	0.152 (0.06)	−0.007 (0.05)	0.013 (−.012, .038)	.302
Stiffness (N/mm)	73 604.989 (21 829)	76 068.224 (22 906)	2463.235 (10 277)	85 041.613 (24 878)	84 445.276 (23 414)	−596.337 (11 533)	3059.6 (−1770.5, 7889.6)	.211
Failure load (N)	3988.445 (1177.0)	4102.143 (1220.2)	113.698 (571.04)	4541.929 (1247.1)	4587.284 (1274.2)	45.355 (344.29)	68.343 (−133.38, 270.07)	.502
HR-pQCT of tibia^a^								
Total vBMD (mg HA/cm^3^)	290.579 (65.15)	292.758 (65.69)	2.179 (9.39)	340.510 (57.95)	340.621 (59.03)	0.112 (5.46)	2.067 (−1.385, 5.519)	.237
Trabecular vBMD (mg HA/cm^3^)	145.133 (51.92)	146.655 (51.15)	1.521 (5.94)	174.207 (32.50)	174.455 (31.65)	0.248 (5.41)	1.274 (−1.344, 3.891)	.335
Cortical vBMD (mg HA/cm^3^)	1004.064 (39.83)	1004.558 (35.78)	0.494 (19.49)	1006.558 (35.29)	1013.600 (32.48)	7.042 (13.31)	−6.548 (−14.365, 1.269)	.099
Cortical area (mm^2^)	304.712 (58.89)	303.867 (58.23)	−0.845 (7.41)	317.516 (53.70)	315.537 (54.09)	−1.979 (4.98)	1.133 (−1.822, 4.089)	.447
Cortical porosity (%)	0.945 (0.81)	1.030 (0.83)	0.085 (0.30)	0.811 (0.63)	0.789 (0.58)	−0.021 (0.23)	0.106 (−.019, .230)	.094
Cortical perimeter (mm)	84.821 (6.64)	84.609 (6.54)	−0.212 (0.67)	84.232 (6.54)	83.889 (6.54)	−0.342 (0.83)	0.130 (−.232, .492)	.476
Cortical thickness (mm)	5.831 (1.15)	5.829 (1.13)	−0.003 (0.18)	6.279 (1.03)	6.219 (1.06)	−0.060 (0.15)	0.057 (−.022, .136)	.152
Cortical pore diameter (mm)	0.194 (0.03)	0.208 (0.04)	0.014 (0.03)	0.222 (0.07)	0.227 (0.07)	0.006 (0.05)	0.008 (−0.012, .028)	.418
Stiffness (N/mm)	193 866.570 (56 132)	197 563.736 (57 580)	3697.167 (11 069)	223 164.371 (48 663)	223 346.240 (48 472)	181.869 (12 434)	3515.3 (−1980.7, 9011.3)	.206
Failure load (N)	10 543.316 (2914.1)	10 710.008 (3013.6)	166.692 (572.15)	11 994.545 (2519.5)	11 999.326 (2539.0)	4.781 (626.01)	161.91 (−117.65, 441.47)	.252
DXA								
Total hip BMD (g/cm^2^)	0.989 (0.19)	1.004 (0.19)	0.016 (0.03)	1.044 (0.16)	1.036 (0.16)	−0.008 (0.04)	0.024 (.009, .039)	.**003**
Femoral neck BMD (g/cm^2^)	0.876 (0.17)	0.871 (0.18)	−0.005 (0.04)	0.889 (0.16)	0.885 (0.17)	−0.004 (0.05)	−0.001 (−.020, .018)	.915
Lumbar spine BMD (g/cm^2^)	1.125 (0.22)	1.160 (0.24)	0.035 (0.06)	1.153 (0.18)	1.165 (0.19)	0.012 (0.04)	0.023 (.000, .045)	.**049**
Trabecular bone score	1.357 (0.13)	1.350 (0.13)	−0.008 (0.08)	1.370 (0.13)	1.370 (0.12)	−0.000 (0.08)	−0.007 (−.042, .028)	.677
Appendicular lean mass index (kg/m^2^)	8.032 (1.31)	7.841 (1.59)	−0.191 (0.88)	8.026 (1.40)	7.930 (1.33)	−0.097 (0.41)	−0.094 (−.385, .196)	.520
Whole-body FMI (kg/m^2^)	8.680 (3.91)	8.582 (4.39)	−0.098 (1.64)	9.471 (5.17)	9.565 (5.09)	0.093 (1.16)	−0.191 (−.804, .422)	.537
Visceral fat area (cm^2^)	93.946 (46.64)	92.728 (53.51)	−1.218 (26.49)	106.489 (69.83)	100.512 (62.23)	−5.977 (24.61)	4.759 (−6.429, 15.948)	.400
Log cytokine (pg/mL)								
Log IL-6	0.430 (0.34)	0.537 (0.35)	0.108 (0.37)	0.334 (0.37)	0.343 (0.32)	0.009 (0.31)	0.099 (−.058, .255)	.214
Log IL-18	2.405 (0.25)	2.320 (0.25)	−0.085 (0.20)	2.257 (0.22)	2.343 (0.18)	0.086 (0.15)	−0.171 (−.251, −.092)	**<**.**001**
Log TNF-α	1.079 (0.20)	1.029 (0.20)	−0.050 (0.13)	0.947 (0.15)	1.031 (0.15)	0.084 (0.16)	−0.133 (−.201, −.065)	**<**.**001**

Results are reported as mean (SD) unless otherwise noted. Bold indicates *P* < .05.

Abbreviations: BMD, bone mineral density; CI, confidence interval; DXA, dual-energy X-ray absorptiometry; FMI, fat mass index; HA, hydroxyapatite; HCV, hepatitis C virus; HR-pQCT, high-resolution peripheral quantitative computed tomography; IL-6, interleukin 6; IL-18, interleukin 18; SD, standard deviation; TNF-α, tumor necrosis factor alpha; vBMD, volumetric bone mineral density.

^a^Total vBMD, trabecular vBMD, stiffness, and failure load were measured at the ultradistal location; all other HR-pQCT variables were measured at the midshaft location.

Between month 0 and month 18, we observed increased mean total hip BMD by DXA (mean change, +0.016 vs −0.008 g/cm^2^; *P* = .003) and mean lumbar spine BMD (mean change, +0.035 vs +0.012 g/cm^2^; *P* = .049) among participants with cured HCV compared to controls. There were no statistically significant differences in mean changes in other DXA measurements between the groups (Table 2, Supplementary Table 1). After adjustment for age, sex, change in ALMI, change in visceral fat area, and smoking, mean month 0–18 differences in DXA measurements between the groups were not statistically significant (Table 3).

**Table 3.** Adjusted Mean Differences in the Change in Bone Measurements From Month 0 to Month 18 Between Participants With and Those Without Hepatitis C Virus Infection

Bone Measurement	Mean Difference Compared to Controls (95% CI)	*P* Value
HR-pQCT of radius^a^		
Total vBMD (mg HA/cm^3^)	−1.769 (−8.663, 5.125)	.611
Trabecular vBMD (mg HA/cm^3^)	1.582 (−4.580, 7.743)	.610
Cortical vBMD (mg HA/cm^3^)	0.585 (−5.163, 6.333)	.840
Cortical area (mm^2^)	0.037 (−1.380, 1.453)	.959
Cortical porosity (%)	−0.040 (−.151, .071)	.473
Cortical perimeter (mm)	−0.212 (−.519, .095)	.173
Cortical thickness (mm)	0.012 (−.060, .083)	.742
Cortical pore diameter (mm)	0.018 (−.013, .048)	.249
Stiffness (N/mm)	5485 (−295, 11265)	.063
Failure load (N)	149.1 (−93.5, 391.8)	.225
HR-pQCT of tibia^a^		
Total vBMD (mg HA/cm^3^)	2.523 (−1.628, 6.674)	.229
Trabecular vBMD (mg HA/cm^3^)	1.966 (−1.268, 5.200)	.229
Cortical vBMD (mg HA/cm^3^)	−6.637 (−16.20, 2.924)	.170
Cortical area (mm^2^)	2.405 (−1.061, 5.871)	.170
Cortical porosity (%)	0.078 (−.061, .216)	.266
Cortical perimeter (mm)	0.240 (−.188, .668)	.266
Cortical thickness (mm)	0.083 (−.008, .173)	.073
Cortical pore diameter (mm)	0.009 (−.014, .032)	.442
Stiffness (N/mm)	1294 (−4954, 7542)	.680
Failure load (N)	48.4 (−265.5, 362.2)	.759
DXA^b^		
Total hip BMD (g/cm^2^)	−0.002 (−.020, .016)	.794
Femoral neck BMD (g/cm^2^)	−0.013 (−.039, .013)	.312
Lumbar spine BMD (g/cm^2^)	0.005 (−.025, .035)	.761
Trabecular bone score	−0.023 (−.066, .019)	.280

All models are adjusted for age, sex, change in appendicular lean mass index, change in visceral fat area, and smoking at month 0.

Abbreviations: BMD, bone mineral density; CI, confidence interval; DXA, dual-energy X-ray absorptiometry; HA, hydroxyapatite; HR-pQCT, high-resolution peripheral quantitative computed tomography; vBMD, volumetric bone mineral density.

^a^Total vBMD, trabecular vBMD, stiffness, and failure load were measured at the ultradistal location; all other HR-pQCT variables were measured at the midshaft location.

^b^Analyses of DXA outcomes were additionally adjusted for a variable indicating that 2 different machines were used by the participant.

When we stratified the cured HCV group by HIV status and repeated the primary adjusted analysis (Supplementary Table 3), we observed several statistically significant differences in mean changes in HR-pQCT measures of the radius, but not tibia. Participants with cured HCV and no HIV (n = 48) had a decrease in radius cortical porosity (adjusted mean difference, −0.127%; *P* = .028), increase in radius stiffness (adjusted mean difference, +8833 N/mm; *P* = .006), increase in radius failure load (adjusted mean difference, +316.0 N; *P* = .016), and increase in tibia cortical porosity (adjusted mean difference, +0.165%; *P* = .022) compared to controls. Participants with cured HCV plus HIV infection (n = 12) had increases in radius cortical porosity (adjusted mean difference, +0.179%; *P* = .027) and radius cortical pore diameter (adjusted mean difference, +0.048 mm; *P* = .043) compared to controls. We observed no differences in DXA measures between the groups stratified by HIV status. Results were similar when change in visceral fat area was replaced with change in FMI (Supplementary Table 2).

### Month 0–18 Changes in Inflammatory Cytokines

Between month 0 and month 18, participants with cured HCV infection had decreases in mean log levels of IL-18 (mean change, −0.085 vs +0.086; mean difference, −0.171; *P* < .001) and TNF-α (mean change, −0.050 vs +0.084; mean difference, −0.133; *P* < .001), but not IL-6 (mean change, +0.108 vs +0.009; mean difference, +0.099; *P* = .2), compared to those without HCV (Table 2, Supplementary Table 1). After adjustment for age, sex, change in visceral fat area, and smoking, the observed decreases in mean log levels of IL-18 (adjusted mean difference, −0.158; *P* = .002) and TNF-α (adjusted mean difference, −0.108; *P* = .009) remained significant for participants with cured HCV versus controls (Table 4). Results were similar when change in visceral fat area was replaced with change in FMI (Supplementary Table 4).

**Table 4.** Adjusted Mean Differences in the Change in Log Cytokine Levels From Month 0 to Month 18 Between Participants With and Without Hepatitis C Virus Infection (Primary Analysis) and Additionally Stratified by HIV Status (Secondary Analysis)

Cytokine	Primary Analysis HIV/HCV and HCV Combined		Secondary Analysis			
			HIV/HCV Only		HCV Only	
	Mean Difference Compared to Controls (95% CI)	*P* Value	Mean Difference Compared to Controls (95% CI)	*P* Value	Mean Difference Compared to Controls (95% CI)	*P* Value
IL-6	0.085 (−.094, .264)	.346	0.185 (−.128, .498)	.241	0.055 (−.141, .250)	.579
IL-18	−0.158 (−.255, −.061)	**.002**	−0.117 (−.286, .053)	.174	−0.170 (−.276, −.064)	.**002**
TNF-α	−0.108 (−.188, −.028)	**.009**	−0.114 (−.254, .026)	.109	−0.106 (−.194, −.018)	.**019**

Data are presented as log cytokine (pg/mL). Bold indicates *P* < .05. All models are adjusted for age, sex, change in visceral fat area, and smoking at month 0.

Abbreviations: CI, confidence interval; HCV, hepatitis C virus; HIV, human immunodeficiency virus; IL-6, interleukin 6; IL-18, interleukin 18; TNF-α, tumor necrosis factor alpha.

When we stratified the cured HCV group by HIV status, we observed no statistically significant differences in mean changes in log IL-18, TNF-α, or IL-6 levels between participants with cured HCV plus HIV infection and those without HCV (Table 4). However, we observed significant decreases in mean log levels of IL-18 (adjusted mean difference, −0.170; *P* = .002) and TNF-α (adjusted mean difference, −0.106; *P* = .019) for participants with cured HCV and no HIV compared to those without HCV (Table 4). Results were similar when change in visceral fat area was replaced with change in FMI (Supplementary Table 4).

## DISCUSSION

We found no significant differences in mean changes in any HR-pQCT measures of the radius or tibia or any BMD measures by DXA from month 0 to month 18 between participants with cured HCV and those without HCV infection after adjustment for age, sex, change in ALMI, change in visceral fat area, and smoking. Participants with cured HCV did have significant decreases in levels of IL-18 and TNF-α, but not IL-6, compared to controls.

There are few data examining the effects of cure of HCV infection on bone, and to our knowledge, none have evaluated the impact of cure on bone microarchitecture or mechanical properties. In a cohort study of 36 noncirrhotic patients with chronic HCV who achieved cure following treatment with pegylated interferon (PEG-IFN) + ribavirin (RBV), lumbar spine and femoral neck BMD measurements 48 weeks after treatment completion were significantly higher compared to baseline [67]. A cohort study of 46 noncirrhotic children with chronic HCV treated with PEG-IFN + RBV, 23 of whom achieved cure, showed significant improvement in BMD by DXA 24 weeks after treatment completion compared to baseline [68]. A cohort study of 30 noncirrhotic patients with chronic HCV treated with PEG-IFN + RBV found that antiviral therapy led to on-treatment increases of lumbar spine and hip BMD by DXA [37]. Among patients with cure (n = 19), most parameters remained highly above baseline values by the end of the 24-week follow-up period, while patients with virological relapse (n = 11) had decreases of BMD. Finally, in a prospective study of 238 people with HIV/HCV coinfection (50% with cirrhosis) treated with antiviral therapy (53.4% with PEG-IFN + RBV + DAA; 34.4% PEG-IFN + RBV; 12.2% sofosbuvir + RBV 12.2%), there was no significant effect of HCV cure (n = 145) on BMD at the lumbar spine and femoral neck or biomarkers of bone remodeling, including soluble receptor activator of nuclear factor-κβ ligand or osteoprotegerin [69].

It remains unclear why bone microarchitecture did not significantly change among participants with HCV infection following cure relative to controls, despite the observed reductions in inflammatory markers. We offer 3 possible reasons for this observation. First, illicit drug use, poor nutrition, and fragility among participants with HCV infection might have contributed to the lack of change in bone quality, irrespective of the effects of HCV cure. Second, the 18-month observation period might not have been long enough to observe changes in bone measurements. The optimal time to examine changes in bone microarchitecture after HCV cure is unknown. We examined bone measurements at month 18 (at least 12 months after cure) because one study evaluating pQCT measures after initiation of Crohn disease therapy found that changes in trabecular volumetric BMD and endocortical bone were evident 12 months after treatment initiation [70, 71]. However, longer follow-up after HCV cure may be necessary to observe changes in bone microarchitecture and strength and should be pursued in future studies. Third, our inclusion of participants with controlled HIV infection on antiretroviral therapy might have prevented us from observing a significant change in bone quality in the cohort of participants with HCV infection. When evaluating results stratified by HIV status, we did observe that participants with cured HCV and no HIV had some improvements in bone microarchitecture, including a decrease in radius cortical porosity and increases in radius stiffness and failure load, compared to controls. The radius bone is less subject to mechanical forces of weight bearing and might be more sensitive to changes in microarchitecture following HCV cure [72 , 73], which may explain why we observed improvements in measurements at the radius but not tibia. These patients also had improvements in cytokine levels, including significant decreases in mean levels of IL-18 and TNF-α over the 18-month observation period. No consistent patterns of improvement in HR-pQCT measurements across the radius and tibia were observed for participants with cured HCV plus HIV infection, and no significant decreases in IL-6, IL-18, or TNF-α were found in this group. It is possible that HIV coinfection might interfere with improvements in bone microarchitecture and mechanical properties following HCV cure. However, these results should be interpreted with caution given the small sample of participants with HIV (n = 12). Future studies evaluating changes in bone quality following HCV cure should account for these considerations.

The control group selected for this study might have affected the study conclusions. To promote balance in demographic characteristics between the study cohorts, we implemented a stratified sampling approach to enroll patients without HCV infection according to sex assigned at birth, race (Black vs not Black), and age category (18–39, 40–59, ≥60 years). However, because of unexpected study participant withdrawals within each group over the study period, the cohort of control participants who completed the study were younger, less commonly reported smoking, and less frequently used illicit drugs. In addition, our study design did not permit enrollment of control participants with HIV infection. These factors might have contributed to changes in bone measurements and inflammatory cytokines over time among control participants that could have affected group differences in measurements under study.

We observed declines in IL-18 and TNF-α following HCV cure. Prior analyses also observed declines in select cytokines after cure. One cohort study of 56 patients with chronic HCV evaluated serum cytokine levels before antiviral therapy and after cure. Significantly lower concentrations of IL-4, IL-9, IL-10, IL-13, and TNF-α were observed after cure compared with pretreatment [74]. A separate analysis of 127 people with HIV/HCV coinfection who received a 12-week course of ledipasvir/sofosbuvir (99 who achieved cure) evaluated the impact of HCV cure on serum IL-6 and soluble tumor necrosis factor receptor I [75]. Significant reductions in soluble tumor necrosis factor receptor I (*P* < .001) were observed among patients who achieved cure. Decreases in systemic inflammation following HCV cure might also have beneficial effects on other organ systems [76].

Our study had several limitations. First, we did not collect duration of HCV infection (since infection date is challenging to ascertain), nutritional status, or some comorbidities (heart failure, thyroid disease) that might affect bone. Further, use of opioids has been associated with low BMD [77, 78], but we did not collect data on use of prescribed or illicit opioids. These factors might be important contributors to bone deficits in this population. Second, we aimed to include 100 participants (50 per group) to have sufficient power to detect group differences in month 0–18 changes in bone measurements and cytokines, but we experienced larger than anticipated withdrawals from each group due to the reasons specified in Figure 1. Third, we did not adjust for multiple testing to preserve power to detect associations given the hypothesis-generating nature of the study. Finally, although advanced hepatic fibrosis/cirrhosis might contribute to bone deficits and cytokine levels, we had too few participants with advanced liver disease to evaluate associations with bone deficits and cytokine levels.

Our study had several strengths. We assessed bone microarchitecture by HR-pQCT, which provides more accurate quantification of trabecular and cortical structure than DXA or conventional pQCT. We measured tibia/radius bone mechanical properties, including axial stiffness and failure load using micro-finite element analysis. We assessed levels of HCV-induced cytokines that can affect osteoblasts and osteoclasts to evaluate changes following HCV cure. Finally, we measured and controlled for important confounding variables, including change in visceral fat area, change in FMI, change in ALMI, and smoking.

In conclusion, we observed no significant changes in bone microarchitecture by HR-pQCT or BMD by DXA in participants with HCV infection from DAA pretreatment through 18 months after DAA initiation compared to those without HCV. Participants with cured HCV had significant decreases in IL-18 and TNF-α, but not IL-6, compared to controls. Future studies should evaluate changes in bone microarchitecture and mechanical properties following HCV cure over longer follow-up to ascertain whether eradication of HCV infection with DAA therapy does indeed reverse the development of bone deficits.

## Supplementary Material

ofaf571_Supplementary_Data

## Supplementary Data

Supplementary materials are available at *Open Forum Infectious Diseases* online. Consisting of data provided by the authors to benefit the reader, the posted materials are not copyedited and are the sole responsibility of the authors, so questions or comments should be addressed to the corresponding author.

## References

[1] Cacoub P, Saadoun D. Extrahepatic manifestations of chronic HCV infection. N Engl J Med 2021; 384:1038–52. 33730456 10.1056/NEJMra2033539

[2] Rouillard S, Lane NE. Hepatic osteodystrophy. Hepatology 2001; 33:301–7. 11124849 10.1053/jhep.2001.20533

[3] Leslie WD, Bernstein CN, Leboff MS; American Gastroenterological Association Clinical Practice Committee. AGA technical review on osteoporosis in hepatic disorders. Gastroenterology 2003; 125:941–66. 12949738 10.1016/s0016-5085(03)01062-x

[4] Raslan HM, Elhosary Y, Ezzat WM, Rasheed EA, Rasheed MA. The potential role of insulin-like growth factor 1, insulin-like growth factor binding protein 3 and bone mineral density in patients with chronic hepatitis C virus in Cairo, Egypt. Trans R Soc Trop Med Hyg 2010; 104:429–32. 20189618 10.1016/j.trstmh.2010.01.012

[5] Lin JC, Hsieh TY, Wu CC, et al Association between chronic hepatitis C virus infection and bone mineral density. Calcif Tissue Int 2012; 91:423–9. 23052227 10.1007/s00223-012-9653-y

[6] Orsini LG, Pinheiro MM, Castro CH, Silva AE, Szejnfeld VL. Bone mineral density measurements, bone markers and serum vitamin D concentrations in men with chronic non-cirrhotic untreated hepatitis C. PLoS One 2013; 8:e81652. 24312334 10.1371/journal.pone.0081652 PMC3842940

[7] Chen CH, Lin CL, Kao CH. Relation between hepatitis C virus exposure and risk of osteoporosis: a nationwide population-based study. Medicine (Baltimore) 2015; 94:e2086. 26632720 10.1097/MD.0000000000002086 PMC5058989

[8] Lo Re V 3rd, Volk J, Newcomb CW, et al Risk of hip fracture associated with hepatitis C virus infection and hepatitis C/human immunodeficiency virus coinfection. Hepatology 2012; 56:1688–98. 22619086 10.1002/hep.25866 PMC3433632

[9] Hansen AB, Omland LH, Krarup H, Obel N, study D. Fracture risk in hepatitis C virus infected persons: results from the DANVIR cohort study. J Hepatol 2014; 61:15–21. 24650694 10.1016/j.jhep.2014.03.007

[10] Mittalhenkle A, Gillen DL, Stehman-Breen CO. Increased risk of mortality associated with hip fracture in the dialysis population. Am J Kidney Dis 2004; 44:672–9. 15384018

[11] Kontulainen SA, Johnston JD, Liu D, Leung C, Oxland TR, McKay HA. Strength indices from pQCT imaging predict up to 85% of variance in bone failure properties at tibial epiphysis and diaphysis. J Musculoskelet Neuronal Interact 2008; 8:401–9. 19147978

[12] Mikolajewicz N, Bishop N, Burghardt AJ, et al HR-pQCT measures of bone microarchitecture predict fracture: systematic review and meta-analysis. J Bone Miner Res 2020; 35:446–59. 31643098 10.1002/jbmr.3901

[13] Weinstein EJ, Carbonari DM, Newcomb CW, et al Abnormal trabecular and cortical bone microarchitecture in chronic hepatitis C infection and associations with select inflammatory cytokines. Open Forum Infect Dis 2025; 12:ofaf102. 40302727 10.1093/ofid/ofaf102 PMC12039487

[14] Nelson DR, Lim HL, Marousis CG, et al Activation of tumor necrosis factor-alpha system in chronic hepatitis C virus infection. Dig Dis Sci 1997; 42:2487–94. 9440625 10.1023/a:1018804426724

[15] Chen TY, Hsieh YS, Wu TT, et al Impact of serum levels and gene polymorphism of cytokines on chronic hepatitis C infection. Transl Res 2007; 150:116–21. 17656331 10.1016/j.trsl.2007.01.007

[16] Sharma A, Chakraborti A, Das A, Dhiman RK, Chawla Y. Elevation of interleukin-18 in chronic hepatitis C: implications for hepatitis C virus pathogenesis. Immunology 2009; 128(1 Suppl):e514–22. 19740312 10.1111/j.1365-2567.2008.03021.x PMC2753952

[17] Shrivastava S, Mukherjee A, Ray R, Ray RB. Hepatitis C virus induces interleukin-1beta (IL-1beta)/IL-18 in circulatory and resident liver macrophages. J Virol 2013; 87:12284–90. 24006444 10.1128/JVI.01962-13 PMC3807883

[18] Radkowski M, Grabarczyk P, Kryczka T, et al Cytokine profile and viral diversity in the early seronegative stage of community-acquired hepatitis C virus (HCV) infection. Sci Rep 2023; 13:20045. 37973814 10.1038/s41598-023-47335-x PMC10654698

[19] Gilbert L, He X, Farmer P, et al Inhibition of osteoblast differentiation by tumor necrosis factor-alpha. Endocrinology 2000; 141:3956–64. 11089525 10.1210/endo.141.11.7739

[20] Li X, Zhou ZY, Zhang YY, Yang HL. IL-6 contributes to the defective osteogenesis of bone marrow stromal cells from the vertebral body of the glucocorticoid-induced osteoporotic mouse. PLoS One 2016; 11:e0154677. 27128729 10.1371/journal.pone.0154677 PMC4851291

[21] Mansoori MN, Shukla P, Kakaji M, et al IL-18BP is decreased in osteoporotic women: prevents inflammasome mediated IL-18 activation and reduces Th17 differentiation. Sci Rep 2016; 6:33680. 27649785 10.1038/srep33680 PMC5030484

[22] Cao X . RANKL-RANK signaling regulates osteoblast differentiation and bone formation. Bone Res 2018; 6:35. 30510840 10.1038/s41413-018-0040-9 PMC6255775

[23] Lee SE, Chung WJ, Kwak HB, et al Tumor necrosis factor-alpha supports the survival of osteoclasts through the activation of Akt and ERK. J Biol Chem 2001; 276:49343–9. 11675379 10.1074/jbc.M103642200

[24] Marahleh A, Kitaura H, Ohori F, et al TNF-alpha directly enhances osteocyte RANKL expression and promotes osteoclast formation. Front Immunol 2019; 10:2925. 31921183 10.3389/fimmu.2019.02925 PMC6923682

[25] Afdhal N, Zeuzem S, Kwo P, et al Ledipasvir and sofosbuvir for untreated HCV genotype 1 infection. N Engl J Med 2014; 370:1889–98. 24725239 10.1056/NEJMoa1402454

[26] Feld JJ, Kowdley KV, Coakley E, et al Treatment of HCV with ABT-450/r-ombitasvir and dasabuvir with ribavirin. N Engl J Med 2014; 370:1594–603. 24720703 10.1056/NEJMoa1315722

[27] Sulkowski MS, Gardiner DF, Rodriguez-Torres M, et al Daclatasvir plus sofosbuvir for previously treated or untreated chronic HCV infection. N Engl J Med 2014; 370:211–21. 24428467 10.1056/NEJMoa1306218

[28] Naggie S, Cooper C, Saag M, et al Ledipasvir and sofosbuvir for HCV in patients coinfected with HIV-1. N Engl J Med 2015; 373:705–13. 26196665 10.1056/NEJMoa1501315 PMC4892372

[29] Osinusi A, Townsend K, Kohli A, et al Virologic response following combined ledipasvir and sofosbuvir administration in patients with HCV genotype 1 and HIV co-infection. JAMA 2015; 313:1232–9. 25706232 10.1001/jama.2015.1373 PMC7780246

[30] Wyles DL, Ruane PJ, Sulkowski MS, et al Daclatasvir plus sofosbuvir for HCV in patients coinfected with HIV-1. N Engl J Med 2015; 373:714–25. 26196502 10.1056/NEJMoa1503153

[31] Sulkowski MS, Eron JJ, Wyles D, et al Ombitasvir, paritaprevir co-dosed with ritonavir, dasabuvir, and ribavirin for hepatitis C in patients co-infected with HIV-1: a randomized trial. JAMA 2015; 313:1223–31. 25706092 10.1001/jama.2015.1328

[32] Feld JJ, Jacobson IM, Hezode C, et al Sofosbuvir and velpatasvir for HCV genotype 1, 2, 4, 5, and 6 infection. N Engl J Med 2015; 373:2599–607. 26571066 10.1056/NEJMoa1512610

[33] American Association for the Study of Liver Diseases and the Infectious Diseases Society of America . HCV Guidance: Recommendations for testing, managing, and treating hepatitis C. **2025**. Available at: http://www.hcvguidelines.org/. Accessed September 17, 2025.

[34] Levey AS, Stevens LA, Schmid CH, et al A new equation to estimate glomerular filtration rate. Ann Intern Med 2009; 150:604–12. 19414839 10.7326/0003-4819-150-9-200905050-00006 PMC2763564

[35] Shahnazarian V, Ramai D, Reddy M, Mohanty S. Hepatitis C virus genotype 3: clinical features, current and emerging viral inhibitors, future challenges. Ann Gastroenterol 2018; 31:541–51. 30174390 10.20524/aog.2018.0281 PMC6102453

[36] Zheng M, Xu J, Feng Z. Association between nonalcoholic fatty liver disease and bone mineral density: Mendelian randomization and mediation analysis. Bone Rep 2024; 22:101785. 39220175 10.1016/j.bonr.2024.101785 PMC11363625

[37] Hofmann WP, Kronenberger B, Bojunga J, et al Prospective study of bone mineral density and metabolism in patients with chronic hepatitis C during pegylated interferon alpha and ribavirin therapy. J Viral Hepat 2008; 15:790–6. 18673425 10.1111/j.1365-2893.2008.01038.x

[38] Isaacson JH, Butler R, Zacharek M, Tzelepis A. Screening with the Alcohol Use Disorders Identification Test (AUDIT) in an inner-city population. J Gen Intern Med 1994; 9(10):550–3. 7823225 10.1007/BF02599279

[39] Skinner HA . The drug abuse screening test. Addict Behav 1982; 7(4):363–71. 7183189 10.1016/0306-4603(82)90005-3

[40] Sterling RK, Patel K, Duarte-Rojo A, et al AASLD practice guideline on blood-based non-invasive liver disease assessments of hepatic fibrosis and steatosis. Hepatology 2025; 81:321–57. 38489523 10.1097/HEP.0000000000000845

[41] Burghardt AJ, Kazakia GJ, Ramachandran S, Link TM, Majumdar S. Age- and gender-related differences in the geometric properties and biomechanical significance of intracortical porosity in the distal radius and tibia. J Bone Miner Res 2010; 25:983–93. 19888900 10.1359/jbmr.091104 PMC3153365

[42] Hildebrand T, Rüegsegger P. A new method for the model-independent assessment of thickness in three-dimensional images. J Microsc 1997; 185:67–75.

[43] Hildebrand T, Laib A, Muller R, Dequeker J, Ruegsegger P. Direct three-dimensional morphometric analysis of human cancellous bone: microstructural data from spine, femur, iliac crest, and calcaneus. J Bone Miner Res 1999; 14:1167–74. 10404017 10.1359/jbmr.1999.14.7.1167

[44] Liu XS, Cohen A, Shane E, et al Bone density, geometry, microstructure, and stiffness: relationships between peripheral and central skeletal sites assessed by DXA, HR-pQCT, and cQCT in premenopausal women. J Bone Miner Res 2010; 25:2229–38. 20499344 10.1002/jbmr.111 PMC3128822

[45] Liu XS, Shane E, McMahon DJ, Guo XE. Individual trabecula segmentation (ITS)-based morphological analysis of microscale images of human tibial trabecular bone at limited spatial resolution. J Bone Miner Res 2011; 26:2184–93. 21557311 10.1002/jbmr.420

[46] Liu XS, Stein EM, Zhou B, et al Individual trabecula segmentation (ITS)-based morphological analyses and microfinite element analysis of HR-pQCT images discriminate postmenopausal fragility fractures independent of DXA measurements. J Bone Miner Res 2012; 27:263–72. 22072446 10.1002/jbmr.562 PMC3290758

[47] Liu XS, Walker MD, McMahon DJ, et al Better skeletal microstructure confers greater mechanical advantages in Chinese-American women versus white women. J Bone Miner Res 2011; 26:1783–92. 21351150 10.1002/jbmr.378 PMC3551974

[48] Liu XS, Wang J, Zhou B, et al Fast trabecular bone strength predictions of HR-pQCT and individual trabeculae segmentation-based plate and rod finite element model discriminate postmenopausal vertebral fractures. J Bone Miner Res 2013; 28:1666–78. 23456922 10.1002/jbmr.1919 PMC3688669

[49] Kelly TL, Wilson KE, Heymsfield SB. Dual energy X-ray absorptiometry body composition reference values from NHANES. PLoS One 2009; 4:e7038. 19753111 10.1371/journal.pone.0007038 PMC2737140

[50] Lebrasseur NK, Achenbach SJ, Melton LJ 3rd, Amin S, Khosla S. Skeletal muscle mass is associated with bone geometry and microstructure and serum insulin-like growth factor binding protein-2 levels in adult women and men. J Bone Miner Res 2012; 27:2159–69. 22623219 10.1002/jbmr.1666 PMC3645866

[51] Baker JF, Davis M, Alexander R, et al Associations between body composition and bone density and structure in men and women across the adult age spectrum. Bone 2013; 53:34–41. 23238122 10.1016/j.bone.2012.11.035 PMC3552077

[52] Choi HS, Kim KJ, Kim KM, et al Relationship between visceral adiposity and bone mineral density in Korean adults. Calcif Tissue Int 2010; 87:218–25. 20631995 10.1007/s00223-010-9398-4

[53] Cohen A, Dempster DW, Recker RR, et al Abdominal fat is associated with lower bone formation and inferior bone quality in healthy premenopausal women: a transiliac bone biopsy study. J Clin Endocrinol Metab 2013; 98:2562–72. 23515452 10.1210/jc.2013-1047 PMC3667251

[54] Gilsanz V, Chalfant J, Mo AO, Lee DC, Dorey FJ, Mittelman SD. Reciprocal relations of subcutaneous and visceral fat to bone structure and strength. J Clin Endocrinol Metab 2009; 94:3387–93. 19531595 10.1210/jc.2008-2422 PMC2741723

[55] Bredella MA, Torriani M, Ghomi RH, et al Determinants of bone mineral density in obese premenopausal women. Bone 2011; 48:748–54. 21195217 10.1016/j.bone.2010.12.011 PMC3073669

[56] Lang T, Cauley JA, Tylavsky F, Bauer D, Cummings S, Harris TB. Computed tomographic measurements of thigh muscle cross-sectional area and attenuation coefficient predict hip fracture: the health, aging, and body composition study. J Bone Miner Res 2010; 25:513–9. 20422623 10.1359/jbmr.090807 PMC3153392

[57] Yerges-Armstrong LM, Miljkovic I, Cauley JA, et al Adipose tissue and volumetric bone mineral density of older Afro-Caribbean men. J Bone Miner Res 2010; 25:2221–8. 20499353 10.1002/jbmr.107 PMC3119489

[58] Sheu Y, Marshall LM, Holton KF, et al Abdominal body composition measured by quantitative computed tomography and risk of non-spine fractures: the Osteoporotic Fractures in Men (MrOS) study. Osteoporos Int 2013; 24:2231–41. 23471565 10.1007/s00198-013-2322-9 PMC3947542

[59] Deere K, Sayers A, Viljakainen HT, et al Distinct relationships of intramuscular and subcutaneous fat with cortical bone: findings from a cross-sectional study of young adult males and females. J Clin Endocrinol Metab 2013; 98:E1041–9. 23533224 10.1210/jc.2013-1272 PMC3752522

[60] Wong AK, Beattie KA, Min K, et al Peripheral quantitative computed tomography-derived muscle density and peripheral magnetic resonance imaging-derived muscle adiposity: precision and associations with fragility fractures in women. J Musculoskelet Neuronal Interact 2014; 14:401–10. 25524965 PMC5092150

[61] Zhang P, Peterson M, Su GL, Wang SC. Visceral adiposity is negatively associated with bone density and muscle attenuation. Am J Clin Nutr 2015; 101:337–43. 25646331 10.3945/ajcn.113.081778 PMC4307204

[62] Tchkonia T, Thomou T, Zhu Y, et al Mechanisms and metabolic implications of regional differences among fat depots. Cell Metab 2013; 17:644–56. 23583168 10.1016/j.cmet.2013.03.008 PMC3942783

[63] Virtue S, Vidal-Puig A. Adipose tissue expandability, lipotoxicity and the metabolic syndrome—an allostatic perspective. Biochim Biophys Acta 2010; 1801:338–49. 20056169 10.1016/j.bbalip.2009.12.006

[64] Oh DY, Morinaga H, Talukdar S, Bae EJ, Olefsky JM. Increased macrophage migration into adipose tissue in obese mice. Diabetes 2012; 61:346–54. 22190646 10.2337/db11-0860 PMC3266418

[65] Cancello R, Tordjman J, Poitou C, et al Increased infiltration of macrophages in omental adipose tissue is associated with marked hepatic lesions in morbid human obesity. Diabetes 2006; 55:1554–61. 16731817 10.2337/db06-0133

[66] Zhang HM, Chen LL, Wang L, et al Macrophage infiltrates with high levels of Toll-like receptor 4 expression in white adipose tissues of male Chinese. Nutr Metab Cardiovasc Dis 2009; 19:736–43. 19356913 10.1016/j.numecd.2008.12.016

[67] Redondo-Cerezo E, Casado-Caballero F, Martin-Rodriguez JL, Hernandez-Quero J, Escobar-Jimenez F, Gonzalez-Calvin JL. Bone mineral density and bone turnover in non-cirrhotic patients with chronic hepatitis C and sustained virological response to antiviral therapy with peginterferon-alfa and ribavirin. Osteoporos Int 2014; 25:1709–15. 24676843 10.1007/s00198-014-2663-z

[68] Megahed A, Salem N, Fathy A, et al Pegylated interferon alpha/ribavirin therapy enhances bone mineral density in children with chronic genotype 4 HCV infection. World J Pediatr 2017; 13:346–52. 28130750 10.1007/s12519-017-0013-x

[69] Carrero A, Berenguer J, Hontanon V, et al Effects of hepatitis C virus (HCV) eradication on bone mineral density in human immunodeficiency virus/HCV-coinfected patients. Clin Infect Dis 2021; 73:e2026–33. 32930720 10.1093/cid/ciaa1396

[70] Thayu M, Leonard MB, Hyams JS, et al Improvement in biomarkers of bone formation during infliximab therapy in pediatric Crohn's disease: results of the REACH study. Clin Gastroenterol Hepatol 2008; 6:1378–84. 19081527 10.1016/j.cgh.2008.07.010

[71] Thayu M, Shults J, Burnham JM, Zemel BS, Baldassano RN, Leonard MB. Gender differences in body composition deficits at diagnosis in children and adolescents with Crohn's disease. Inflamm Bowel Dis 2007; 13:1121–8. 17427245 10.1002/ibd.20149 PMC2705771

[72] Mikkola TM, Sipila S, Rantanen T, et al Genetic and environmental influence on structural strength of weight-bearing and non-weight-bearing bone: a twin study. J Bone Miner Res 2008; 23:492–8. 18072876 10.1359/jbmr.071205

[73] Kelley JC, Stettler-Davis N, Leonard MB, et al Effects of a randomized weight loss intervention trial in obese adolescents on tibia and radius bone geometry and volumetric density. J Bone Miner Res 2018; 33:42–53. 28884881 10.1002/jbmr.3288 PMC8527854

[74] Radmanic L, Bodulic K, Simicic P, Vince A, Lepej SZ. The effect of treatment-induced viral eradication on cytokine and growth factor expression in chronic hepatitis C. Viruses 2022; 14:1613. 35893679 10.3390/v14081613 PMC9394470

[75] Karimi-Sari H, Piggott DA, Scully EP, et al Changes in inflammatory cytokines after chronic hepatitis C treatment among people living with HIV. Open Forum Infect Dis 2024; 11:ofad623. 38192382 10.1093/ofid/ofad623 PMC10773550

[76] Morishima C, Shiffman ML, Dienstag JL, et al Reduction in hepatic inflammation is associated with less fibrosis progression and fewer clinical outcomes in advanced hepatitis C. Am J Gastroenterol 2012; 107:1388–98. 22688849 10.1038/ajg.2012.137 PMC3865099

[77] Daniell HW . Opioid osteoporosis. Arch Intern Med 2004; 164:338; author reply 338. 10.1001/archinte.164.3.338-a 14769633

[78] Krishnamoorthy S, Li GH, Ho KS, et al Illicit drug use is associated with lower bone mineral density and bone strength. Osteoporos Sarcopenia 2023; 9:88–93. 37941531 10.1016/j.afos.2023.09.001 PMC10628013

