# Supplementary material for: Changes in Bone Microarchitecture and Inflammatory Cytokines After Cure of Chronic Hepatitis C Infection With Direct-Acting Antiviral Therapy
Source: Open Forum Infect Dis. 2025 Sep 15;12(9):ofaf571. doi: 10.1093/ofid/ofaf571 (PMC12465109; doi:10.1093/ofid/ofaf571)
Supplement: ofaf571_Supplementary_Data [file ofaf571_supplementary_data.docx]

**Supplementary Appendix**

**Changes in Bone Microarchitecture and Inflammatory Cytokines after Cure of Chronic Hepatitis C Infection With Direct-Acting Antiviral Therapy**

Vincent Lo Re III, Dean M. Carbonari, Craig W. Newcomb, Jessie Torgersen, Erica J. Weinstein, Shanae M. Smith, Katherine L. Brecker, X. Sherry Liu, Jay R. Kostman, Stacey Trooskin, Rebecca A. Hubbard, Joshua F. Baker, Babette S. Zemel, Mary B. Leonard

**Table of Contents**

**Supplemental Table 1.** Number (percent) of missing values for the unadjusted mean changes in HR-pQCT, DXA, and laboratory measurements from Month 0 to Month 18 in participants with and without hepatitis C virus reported in Table 2……………………………………………………………….3

**Supplemental Table 2.** Adjusted mean differences (95% confidence intervals) in the change in bone measurements from Month 0 to Month 18 between participants with and without hepatitis C virus infection (primary analysis) and additionally stratified by HIV status (secondary analysis). All models are adjusted for age, sex, change in appendicular lean mass index, change in fat mass index, and smoking at Month 0………………………………………………………………………………5

**Supplemental Table 3.** Adjusted mean differences (95% confidence intervals) in the change in bone measurements from Month 0 to Month 18 among participants with hepatitis C virus infection stratified by HIV status. All models are adjusted for age, sex, change in appendicular lean mass index, change in visceral fat area, and smoking at Month 0……………………………………………..7

**Supplemental Table 4.** Adjusted mean differences (95% confidence intervals) in the change in log cytokine levels from Month 0 to Month 18 between participants with and without hepatitis C virus infection (primary analysis) and additionally stratified by HIV status (secondary analysis). All models are adjusted for age, sex, change in fat mass index, and smoking at Month 0………………………..8

# **Supplemental Table 1**. Number (percent) of missing values for the unadjusted mean changes in HR-pQCT, DXA, and laboratory measurements from Month 0 to Month 18 in participants with and without hepatitis C virus reported in Table 2.

| **Missing (n, %)** | **Participants with HCV (n=40)** | **Participants without HCV (n=48)** |
| --- | --- | --- |
| **HR-pQCT of Radius**^a^ |  |  |
| Total vBMD (mg HA/cm^3^) | 3 (7.5%) | 2 (4.2%) |
| Trabecular vBMD (mg HA/cm^3^) | 3 (7.5%) | 2 (4.2%) |
| Cortical vBMD (mg HA/cm^3^) | 2 (5.0%) | 3 (6.3%) |
| Cortical area (mm^2^) | 2 (5.0%) | 3 (6.3%) |
| Cortical porosity (%) | 2 (5.0%) | 3 (6.3%) |
| Cortical perimeter (mm) | 2 (5.0%) | 3 (6.3%) |
| Cortical thickness (mm) | 2 (5.0%) | 3 (6.3%) |
| Cortical pore diameter (mm) | 2 (5.0%) | 3 (6.3%) |
| Stiffness (N/mm) | 3 (7.5%) | 2 (4.2%) |
| Failure load (N) | 3 (7.5%) | 2 (4.2%) |
| **HR-pQCT of Tibia**^a^ |  |  |
| Total vBMD (mg HA/cm^3^) | 7 (17.5%) | 6 (12.5%) |
| Trabecular vBMD (mg HA/cm^3^) | 7 (17.5%) | 6 (12.5%) |
| Cortical vBMD (mg HA/cm^3^) | 7 (17.5%) | 10 (20.8%) |
| Cortical area (mm^2^) | 7 (17.5%) | 10 (20.8%) |
| Cortical porosity (%) | 7 (17.5%) | 10 (20.8%) |
| Cortical perimeter (mm) | 7 (17.5%) | 10 (20.8%) |
| Cortical thickness (mm) | 7 (17.5%) | 10 (20.8%) |
| Cortical pore diameter (mm) | 7 (17.5%) | 10 (20.8%) |
| Stiffness (N/mm) | 7 (17.5%) | 6 (12.5%) |
| Failure load (N) | 7 (17.5%) | 6 (12.5%) |
| **DXA** |  |  |
| Total hip BMD (g/cm^2^) | 0 (0%) | 2 (4.2%) |
| Femoral neck BMD (g/cm^2^) | 0 (0%) | 2 (4.2%) |
| Lumbar spine BMD (g/cm^2^) | 3 (7.5%) | 4 (8.3%) |
| Trabecular bone score | 2 (5.0%) | 2 (4.2%) |
| Appendicular lean mass index (kg/m^2^) | 3 (7.5%) | 2 (4.2%) |
| Whole body fat mass index (kg/m^2^) | 3 (7.5%) | 2 (4.2%) |
| Visceral fat area (cm^2^) | 3 (7.5%) | 2 (4.2%) |
| **Log cytokine (pg/mL)** |  |  |
| Log interleukin 6 | 7 (17.5%) | 6 (12.5%) |
| Log interleukin 18 | 7 (17.5%) | 5 (10.4%) |
| Log tumor necrosis factor α | 7 (17.5%) | 5 (10.4%) |

Abbreviations: DXA=dual-energy x-ray absorptiometry; HA=hydroxyapatite; HCV=hepatitis C virus; HR-pQCT= high-resolution peripheral quantitative computed tomography; SD=standard deviation; vBMD=volumetric bone mineral density

^a^ Total volumetric BMD, trabecular volumetric BMD, stiffness, and failure load were measured at the ultradistal location; all other HR-pQCT variables measured at the midshaft location.

# **Supplemental Table 2**. Adjusted mean differences (95% confidence intervals) in the change in bone measurements from Month 0 to Month 18 between participants with and without hepatitis C virus infection (primary analysis) and additionally stratified by HIV status (secondary analysis). All models are adjusted for age, sex, change in appendicular lean mass index, change in fat mass index, and smoking at Month 0.

|  | **Primary Analysis** | | **Secondary Analysis** | | | |
| --- | --- | --- | --- | --- | --- | --- |
|  | **HIV/HCV & HCV Combined**  **(n=40)** | | **HIV/HCV Only**  **(n=12)** | | **HCV Only**  **(n=28)** | |
| **Bone Measurement** | **Mean Difference Compared to Controls**  **(95% CI)** | ***P* Value** | **Mean Difference Compared to Controls**  **(95% CI)** | ***P* Value** | **Mean Difference Compared to Controls**  **(95% CI)** | ***P* Value** |
| **HR-pQCT of Radius**^a^ |  |  |  |  |  |  |
| Total vBMD (mg HA/cm^3^) | -1.722 (-8.598, 5.154) | 0.619 | -10.29 (-20.66, 0.088) | 0.052 | 1.715 (-5.710, 9.139) | 0.647 |
| Trabecular vBMD (mg HA/cm^3^) | 1.473 (-4.671, 7.617) | 0.634 | -2.573 (-12.06, 6.912) | 0.590 | 3.097 (-3.690, 9.884) | 0.366 |
| Cortical vBMD (mg HA/cm^3^) | 0.515 (-5.201, 6.231) | 0.858 | 4.012 (-4.950, 12.97) | 0.375 | -0.823 (-7.120, 5.474) | 0.795 |
| Cortical area (mm^2^) | -0.082 (-1.445, 1.281) | 0.905 | -0.126 (-2.278, 2.026) | 0.908 | -0.065 (-1.577, 1.447) | 0.932 |
| Cortical porosity (%) | -0.041 (-0.148, 0.065) | 0.441 | 0.158 (0.001, 0.315) | **0.049** | -0.118 (-0.228, -0.007) | **0.037** |
| Cortical perimeter (mm) | -0.197 (-0.503, 0.110) | 0.205 | -0.270 (-0.753, 0.214) | 0.270 | -0.169 (-0.508, 0.171) | 0.326 |
| Cortical thickness (mm) | 0.007 (-0.062, 0.075) | 0.845 | 0.017 (-0.092, 0.125) | 0.757 | 0.003 (-0.073, 0.079) | 0.940 |
| Cortical pore diameter (mm) | 0.018 (-0.012, 0.049) | 0.230 | 0.048 (0.001, 0.095) | **0.044** | 0.007 (-0.026, 0.040) | 0.676 |
| Stiffness (N/mm) | 5525 (-240, 11,290) | 0.060 | -2844 (-11,442, 5754) | 0.512 | 8883 (2730, 15,035) | **0.005** |
| Failure load (N) | 133.4 (-106.5, 373.4) | 0.271 | -302.8 (-651.4, 45.8) | 0.088 | 308.4 (59.0, 557.9) | **0.016** |
| **HR-pQCT of Tibia**^a^ |  |  |  |  |  |  |
| Total vBMD (mg HA/cm^3^) | 2.657 (-1.439, 6.752) | 0.200 | -0.849 (-7.366, 5.669) | 0.796 | 3.943 (-0.534, 8.420) | 0.083 |
| Trabecular vBMD (mg HA/cm^3^) | 1.930 (-1.280, 5.141) | 0.234 | 2.909 (-2.267, 8.085) | 0.266 | 1.571 (-1.984, 5.127) | 0.381 |
| Cortical vBMD (mg HA/cm^3^) | -6.846 (-16.33, 2.640) | 0.154 | -5.958 (-21.16, 9.245) | 0.436 | -7.156 (-17.57, 3.262) | 0.175 |
| Cortical area (mm^2^) | 2.358 (-1.082, 5.799) | 0.176 | 1.185 (-4.316, 6.686) | 0.668 | 2.768 (-1.002, 6.537) | 0.147 |
| Cortical porosity (%) | 0.077 (-0.055, 0.209) | 0.249 | -0.136 (-0.336, 0.063) | 0.177 | 0.151 (0.015, 0.288) | **0.031** |
| Cortical perimeter (mm) | 0.227 (-0.199, 0.653) | 0.291 | 0.212 (-0.471, 0.894) | 0.538 | 0.232 (-0.235, 0.700) | 0.324 |
| Cortical thickness (mm) | 0.079 (-0.011, 0.169) | 0.083 | 0.042 (-0.102, 0.185) | 0.565 | 0.092 (-0.006, 0.191) | 0.066 |
| Cortical pore diameter (mm) | 0.008 (-0.015, 0.030) | 0.498 | -0.015 (-0.051, 0.020) | 0.387 | 0.016 (-0.008, 0.040) | 0.198 |
| Stiffness (N/mm) | 1522 (-4698, 7743) | 0.627 | -807 (-10,824, 9211) | 0.873 | 2377 (-4505, 9259) | 0.493 |
| Failure load (N) | 59.2 (-253.1, 371.6) | 0.706 | -53.8 (-556.9, 449.4) | 0.832 | 100.7 (-245.0, 446.3) | 0.563 |
| **DXA**^b^ |  |  |  |  |  |  |
| Total hip BMD (g/cm^2^) | 0.001 (-0.017, 0.019) | 0.911 | -0.001 (-0.029, 0.028) | 0.965 | 0.001 (-0.018, 0.021) | 0.882 |
| Femoral neck BMD (g/cm^2^) | -0.010 (-0.037, 0.016) | 0.430 | -0.014 (-0.056, 0.027) | 0.499 | -0.010 (-0.037, 0.018) | 0.490 |
| Lumbar spine BMD (g/cm^2^) | 0.003 (-0.027, 0.033) | 0.851 | -0.040 (-0.085, 0.004) | 0.077 | 0.014 (-0.016, 0.045) | 0.357 |
| Trabecular bone score | -0.035 (-0.077, 0.007) | 0.099 | -0.078 (-0.143, -0.014) | **0.018** | -0.024 (-0.067, 0.019) | 0.265 |

Bold indicates *P* <0.05.

Abbreviations: DXA=dual-energy x-ray absorptiometry; HA=hydroxyapatite; HCV=hepatitis C virus; HR-pQCT= high-resolution peripheral quantitative computed tomography; vBMD=volumetric bone mineral density

^a^ Total volumetric BMD, trabecular volumetric BMD, stiffness, and failure load were measured at the ultradistal location; all other HR-pQCT variables measured at the midshaft location.

^b^ Analyses of DXA outcomes were additionally adjusted for a variable indicating that two different machines were used by the participant.

# **Supplemental Table 3**. Adjusted mean differences (95% confidence intervals) in the change in bone measurements from Month 0 to Month 18 among participants with hepatitis C virus infection stratified by HIV status. All models are adjusted for age, sex, change in appendicular lean mass index, change in visceral fat area, and smoking at Month 0.

|  | **HIV/HCV Only** | | **HCV Only** | |
| --- | --- | --- | --- | --- |
| **Bone Measurement** | **Mean Difference Compared to Controls**  **(95% CI)** | ***P* Value** | **Mean Difference Compared to Controls**  **(95% CI)** | ***P* Value** |
| **HR-pQCT of Radius**^a^ |  |  |  |  |
| Total vBMD (mg HA/cm^3^) | -9.837 (-20.05, 0.380) | 0.059 | 1.672 (-5.823, 9.166) | 0.658 |
| Trabecular vBMD (mg HA/cm^3^) | -2.207 (-11.54, 7.123) | 0.639 | 3.198 (-3.646, 10.04) | 0.355 |
| Cortical vBMD (mg HA/cm^3^) | 3.971 (-4.863, 12.81) | 0.373 | -0.753 (-7.085, 5.578) | 0.813 |
| Cortical area (mm^2^) | 0.347 (-1.844, 2.537) | 0.753 | -0.086 (-1.656, 1.484) | 0.914 |
| Cortical porosity (%) | 0.179 (0.021, 0.336) | **0.027** | -0.127 (-0.240, -0.014) | **0.028** |
| Cortical perimeter (mm) | -0.271 (-0.745, 0.203) | 0.258 | -0.188 (-0.528, 0.152) | 0.273 |
| Cortical thickness (mm) | 0.039 (-0.071, 0.149) | 0.482 | 0.001 (-0.078, 0.080) | 0.978 |
| Cortical pore diameter (mm) | 0.048 (0.002, 0.094) | **0.043** | 0.006 (-0.027, 0.039) | 0.726 |
| Stiffness (N/mm) | -2365 (-10840, 6109) | 0.580 | 8833 (2616, 15049) | **0.006** |
| Failure load (N) | -242.2 (-591.8, 107.4) | 0.172 | 316.0 (59.6, 572.5) | **0.016** |
| **HR-pQCT of Tibia**^a^ |  |  |  |  |
| Total vBMD (mg HA/cm^3^) | -1.309 (-7.683, 5.064) | 0.683 | 3.988 (-0.521, 8.496) | 0.082 |
| Trabecular vBMD (mg HA/cm^3^) | 2.999 (-2.051, 8.049) | 0.240 | 1.571 (-2.002, 5.143) | 0.383 |
| Cortical vBMD (mg HA/cm^3^) | -5.312 (-20.24, 9.616) | 0.479 | -7.113 (-17.59, 3.360) | 0.179 |
| Cortical area (mm^2^) | 1.381 (-4.021, 6.783) | 0.611 | 2.773 (-1.017, 6.563) | 0.149 |
| Cortical porosity (%) | -0.166 (-0.366, 0.034) | 0.102 | 0.165 (0.025, 0.306) | **0.022** |
| Cortical perimeter (mm) | 0.203 (-0.466, 0.871) | 0.546 | 0.254 (-0.215, 0.723) | 0.284 |
| Cortical thickness (mm) | 0.042 (-0.099, 0.182) | 0.554 | 0.097 (-0.001, 0.196) | 0.053 |
| Cortical pore diameter (mm) | -0.017 (-0.052, 0.017) | 0.322 | 0.018 (-0.006, 0.042) | 0.138 |
| Stiffness (N/mm) | -942 (-10693, 8810) | 0.848 | 2148 (-4750, 9046) | 0.536 |
| Failure load (N) | -60.1 (-550.0, 429.8) | 0.807 | 89.8 (-256.8, 436.4) | 0.606 |
| **DXA**^b^ |  |  |  |  |
| Total hip BMD (g/cm^2^) | -0.007 (-0.035, 0.020) | 0.603 | -0.001 (-0.020, 0.018) | 0.914 |
| Femoral neck BMD (g/cm^2^) | -0.019 (-0.059, 0.021) | 0.340 | -0.012 (-0.039, 0.016) | 0.400 |
| Lumbar spine BMD (g/cm^2^) | -0.031 (-0.075, 0.013) | 0.166 | 0.015 (-0.016, 0.046) | 0.325 |
| Trabecular bone score | -0.046 (-0.111, 0.019) | 0.159 | -0.017 (-0.062, 0.028) | 0.459 |

Bold indicates *P* <0.05.

Abbreviations: DXA=dual-energy x-ray absorptiometry; HA=hydroxyapatite; HCV=hepatitis c virus; HR-pQCT=high-resolution peripheral quantitative computed tomography; vBMD=volumetric bone mineral density

^a^ Total volumetric BMD, trabecular volumetric BMD, stiffness, and failure load were measured at the ultradistal location; all other HR-pQCT variables measured at the midshaft location.

^b^ Analyses of DXA outcomes were additionally adjusted for a variable indicating that two different machines were used by the participant.

# **Supplemental Table 4**. Adjusted mean differences (95% confidence intervals) in the change in log cytokine levels from Month 0 to Month 18 between participants with and without hepatitis C virus infection (primary analysis) and additionally stratified by HIV status (secondary analysis). All models are adjusted for age, sex, change in fat mass index, and smoking at Month 0.

|  | **Primary Analysis** | | **Secondary Analysis** | | | |
| --- | --- | --- | --- | --- | --- | --- |
|  | **HIV/HCV & HCV Combined**  **(n=40)** | | **HIV/HCV Only**  **(n=12)** | | **HCV Only**  **(n=28)** | |
| **Log Cytokine** | **Mean Difference Compared to Controls**  **(95% CI)** | ***P* Value** | **Mean Difference Compared to Controls**  **(95% CI)** | ***P* Value** | **Mean Difference Compared to Controls**  **(95% CI)** | ***P* Value** |
| Interleukin 6 | 0.085 (-0.098, 0.268) | 0.357 | 0.194 (-0.125, 0.513) | 0.229 | 0.053 (-0.146, 0.252) | 0.596 |
| Interleukin 18 | -0.166 (-0.263, -0.068) | **0.001** | -0.127 (-0.298, 0.043) | 0.141 | -0.177 (-0.283, -0.070) | **0.002** |
| Tumor necrosis factor α | -0.108 (-0.189, -0.027) | **0.010** | -0.113 (-0.255, 0.029) | 0.116 | -0.107 (-0.195, -0.018) | **0.019** |

Bold indicates *P* <0.05.

Abbreviations: CI=confidence interval; HCV=hepatitis C virus
